# Failure Analysis of Ni-8YSZ Electrode under Reoxidation Based on the Real Microstructure

**DOI:** 10.3390/ma17184599

**Published:** 2024-09-19

**Authors:** Sen Yang, Zhipeng Chen, Hongye Zhang, Jinzhi Li, Xiang Zhao, Wenqian Hao, Jiamiao Xie, Fenghui Wang

**Affiliations:** 1 Bio-Inspired and Advanced Energy Research Center, Department of Engineering Mechanics, Northwestern Polytechnical University, Xi’an 710072, Chinazhy8528@mail.nwpu.edu.cn (H.Z.);; 2 Songshan Lake Materials Laboratory, Dongguan 523808, China; 3 School of Aerospace Engineering, North University of China, Taiyuan 030051, China

**Keywords:** nano-computed tomography, Ni-8YSZ real microstructure, reoxidation, failure probability

## Abstract

During the operation of solid oxide fuel cells (SOFCs), the Ni-8YSZ anodes are subjected to thermal mismatch and reoxidation, accompanied by the risk of damage and failure. These damages and failures are generally induced by small defects at the microscopic level, leading to the degradation of the structural bearing capacity. Therefore, the distribution and quantification of the stresses in the real microstructure of Ni-8YSZ electrodes is essential. In this study, the real Ni-8YSZ microstructure was reconstructed based on nano-computed tomography, and the stress distribution of the real microstructure was analyzed based on the finite element method under reoxidation and different operating temperatures. The failure probability of 8YSZ at different degrees of reoxidation was evaluated according to the Weibull method, and the amount of damaged 8YSZ elements was statistically counted. The study results indicate a high level of stress in the thin necks and relatively sharp areas of the microstructure. The 8YSZ has a high failure probability at a reoxidation extent of 5–10%.

## 1. Introduction

Solid oxide fuel cells (SOFCs) are promising energy conversion devices that can convert high-quality fuel to electricity and will play an irreplaceable role in the integration of renewable energy resources in the future [1,2]. Porous Ni-8YSZ is the most widely used SOFC anode material due to its relatively low cost, high catalytic activity, and excellent electrical conductivity. Nevertheless, reoxidation occurs during the sudden start–stop of the SOFC, the leakage of the sealing device, and high fuel utilization, while Ni and NiO_x_ convert to each other [3], and the cycle of reduction and oxidation causes irreversible dimensional changes, resulting in the mechanical damage (cracking and delamination) of the SOFC electrolyte and fuel electrodes. Furthermore, the cracking and delamination change the transport path of the ions and electrons and increase the polarization resistance of the electrode [4,5,6]. According to previous studies, the degradation rate of the SOFC anode after redox cycling exceeds 50 times compared to the standard operation [7].

In recent years, several investigations have been conducted to improve the structural integrity of SOFCs by incorporating the known physical and material parameters to predict oxidation-induced stress [8,9,10]. When the degree of anodic oxidation reaches 40–50% and the oxidation strain is 0.1–0.2%, the electrodes and electrolytes of the SOFC are subject to failure. Shang et al. [11] predicted the failure probability of anodes by establishing an oxidation-graded induced stress model, and the failure probability was maximum when the oxidation degree reached 62–72% and the oxidized graded zone was thicker. Wang et al. [12] considered non-uniform and uniform oxidation, and revealed that the maximum principal stress on the anode was five times greater in non-uniform oxidation than in uniform oxidation. However, these macroscopic numerical modeling studies assumed Ni/NiO–8YSZ as an isotropic material, which only varies the mechanical parameters of the oxide layer depending on the degree of oxidation, and did not take into account the interaction between Ni and 8YSZ in the anode. Hence, separating the materials in each part of the SOFC anode for calculations enables a more in-depth understanding of the mechanisms of destruction or degradation.

The implementation of focused ion beam–scanning electron microscopy (FIB-SEM) or X-ray tomography enables the reconstruction of the real 3D microstructure of the SOFC electrodes to precisely determine the distribution of the individual materials in the electrodes and to quantify the electrochemical or mechanical parameters [13,14,15,16,17]. On the one hand, the representative volume elements (RVEs) of the electrode were quantified to evaluate its degradation upon redox cycling. The three-phase boundary (TPB) decreases dramatically (4.90 to 1.06 µm^−2^) and polarization resistance (0.13 to 0.25 Ω·cm^2^) increases after redox [18,19], and these parameters are indicative of the rapid degradation of the electrochemical performance. Shearing et al. [20] demonstrated through 3D reconstruction that the original nickel contact area with the pores decreased significantly from 0.310 to 0.027 µm^2^·µm^−3^ when nickel was oxidized to NiO. In situ single-particle nickel oxidation research has indicated that, when nickel is in contact with oxygen, the low-coordination corners are the active sites of the oxidation reaction [21]. On the other hand, the mechanical simulation of the microstructure of the fuel cell through the finite element analysis (FEA) method could more intuitively understand the stress distribution of each material on the electrodes and accurately predict the location of the electrodes to be damaged. Clague et al. [22] and Kim et al. [14] obtained the real microstructure of the SOFC anode by the FIB-SEM technique, and the thermal stress distribution of the anode during operation was simulated by FEA. The results indicated that the stresses at the interface of NiO/Ni-8YSZ would exceed the failure strength of the material. Xiang et al. [23] investigated the temperature dependence of mechanical parameters and predicted the failure probability of the anode real microstructure under tension and compression. Toros [24] utilized 3D commercial software to randomly generate the microstructures of Ni-8YSZ electrodes to investigate the oxidation rate and stress evolution of electrodes exposed to oxygen. However, there are fewer studies on the stress distribution of the real microstructures under reoxidation conditions. In addition, the generated microstructures were too simple compared to the real microstructure, and thermal stresses were neglected.

In summary, the modeling study of SOFC anodes lacks the simulation of their stress distribution under the coupling of reoxidation and thermal stresses in a real microstructure. In this study, based on nano-X-ray tomography characterization, the real microstructure of the reduced anode was extracted and FEA simulation was implemented. The damage-prone location and failure probability of the electrode in the condition of reoxidation were obtained. This investigation aims to deepen the understanding of the microstructure evolution during reoxidation, which is beneficial for improving the cell durability and structural integrity of the SOFC.

## 2. Experiment by Nano-CT

### 2.1. Sample Preparation

**Figure 1:**
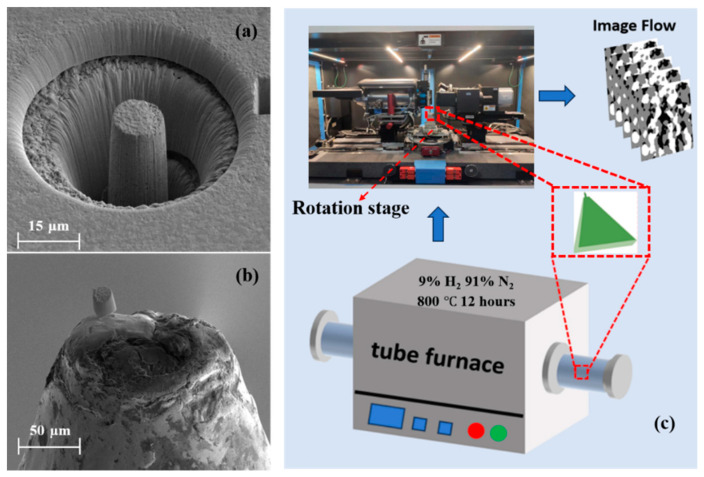
Schematic diagram of the experimental procedure. (**a**) Microcylinder milled by Fib; (**b**) Microcylinder Pt-welded to a polished flat surface; (**c**) Sample reduction reaction and nano-CT scanning of the sample.

The anode of SOFC (NiO-8YSZ) was fabricated by the Ningbo Institute of Materials Technology and Engineering, Chinese Academy of Sciences (Ningbo, China). The anode consists of nickel oxide (NiO) and 8 mol% yttria-stabilized zirconia (8YSZ); the wt % of the NiO and 8YSZ in the anode is 56:44. Subsequently, the sample was placed in the tube furnace (OTF-1200X) and reduced in a 9% H_2_ and 91% N_2_ gas mixture at 800 °C for 12 h to convert the nickel oxide to nickel completely. One corner of the sample was polished to a relatively flat surface of approximately 200 µm with a metallographic sample polishing machine (PG-1A) to facilitate the subsequent welding, and the polished sample was arranged in an ultrasonic cleaner (Derui DR-LQ20, Shenzhen, China) for the ultrasonic cleaning treatment to clean the surface and impurities that might be embedded in the internal pores of the electrodes. Then, the cleaned sample was milled with a focused ion beam (Helios G4 CX, Thermo Fisher Scientific, Waltham, MA, USA) to make the sample into a microcylinder with a diameter of 10 μm and a height of 15 μm. The microcylinder was next cut down and Pt-welded to the flat surface, as shown in Figure 1a,b, which is a size that enabled global scanning with a higher resolution in the nano-CT. The prepared sample was fixed on a 360-degree rotation stage and scanned with the nano-CT (Zeiss Xradia 810 Versa, Oberkochen, Germany). The resolution was set to 64 nm. The acquired dataset consisted of approximately 900–1000 high-quality 2D tomographic images. The experimental procedure is displayed in Figure 1c.

### 2.2. Three-Phase Segmentation and 3D Reconstruction

**Figure 2:**
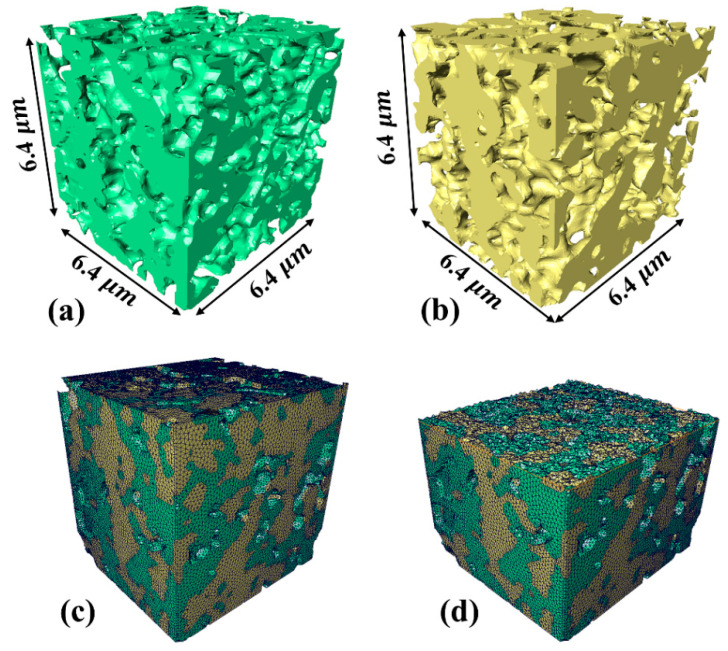
Three-phase segmentation and mesh generation: (**a**) Nickel phase; (**b**) 8YSZ phase; (**c**) Triangular surface mesh; (**d**) Tetra mesh.

Three-phase segmentation is a prerequisite for the subsequent 3D microstructure reconstruction. Consequently, the accurate segmentation and quantification of the three phases (metallic phase Ni/NiO, ceramic phase 8YSZ, and pore phase) is crucial. The tomography raw image dataset was imported into Avizo (Thermo Scientific Amira-Avizo3D 2022.2) and filtered to reduce the noise and normalize the grayscale [25]. The processed image sequences were divided into three-phase boundaries by a 3D watershed algorithm based on a grayscale gradient [26,27]. Subsequently, this resulted in three-phase labels in Avizo, which were regular cubic grids with the same dimensions as the 2D stacking image volume. Overlaying each voxel in the labels of each phase obtained the 3D volume space. The final 3D reconstructed size of the RVEs was 6.4 × 6.4 × 6.4 µm^3^, and the Ni and 8YSZ phases are demonstrated in Figure 2a,b. The triangular surface mesh was generated in Avizo for the segmented Ni and 8YSZ phases, as shown in Figure 2c. Due to the relatively poor quality of the directly generated surface mesh, the aspect ratio, dihedral angle, and tetra quality of the surface mesh were required to be manually modified to improve the quality of the final generated tetrahedral volume mesh; the specific modification requirements can be found in the Avizo Users Guide. Eventually, the tetrahedral volume mesh was generated, which is illustrated in Figure 2d, and the export of the (.inp) format file was imported into Abaqus (Dassault Systèmes, Abaqus/CAE, 2020) for the FEA simulation. This (.inp) file contained node position information and volume mesh matching information for the Ni and 8YSZ.

## 3. Reoxidation Model

### 3.1. Reoxidation Strain

Bedworth and Pilling proposed that the oxidizing stress derives from the volume difference between the metal oxide and the consumed metal. The volume ratio of the metal oxide to the consumed metal is stated as the Pilling–Bedworth ratio, as shown in Equation (1):

(1) $$PBR=\frac{V_{p}}{N_{m}V_{met}}$$

where $V_{p}$ is the molar volume of the oxide, and $N_{m}$ and $V_{met}$ are the number of metal atoms in one unit oxide and molar volume of the metal, respectively. The complete oxidation strain of nickel in the anode as an isotropic material can be expressed as follows:

(2) $$\varepsilon^{ox}=\sqrt[{3}]{PBR}-1$$

The *PBR* of the nickel is 1.65 [28]. Nevertheless, some oxidation experiments have deviated from the theoretical *PBR* values [29,30]. In this study, theoretical *PBR* values were utilized to calculate the oxidative stresses.

### 3.2. Oxidation Kinetics

**Figure 3:**
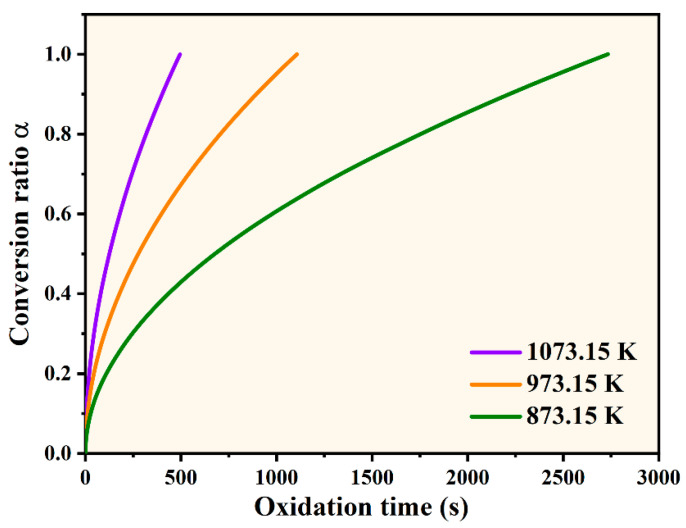
Oxidative conversion of α vs. time.

The kinetic process of nickel oxidation at elevated temperatures is divided into two main stages. At temperatures below 700 °C, the oxidation of nickel is mainly controlled by diffusion along the grain boundaries. When the temperature is elevated to 700–1000 °C, the oxidation of nickel becomes a transition state of the grain boundary and nickel lattice diffusion [31]. In addition, the shape, size, rate of heating, and oxidizing atmosphere of nickel all affect the oxidation process of nickel. However, when processing the experimental data, the oxidation rate was characterized by oxidation using traditional kinetic methods. The oxidation conversion ratio of nickel α is described by Equation (3) [32], as follows:

(3) $$\frac{d\alpha }{dt}=K(T)f(\alpha )$$

where f(α) is the solid-phase reaction mechanism function, f(α) takes 2/α of the parabolic law [33], and the reaction rate K (T) can be expressed as the Arrhenius mode, as follows:

(4) $$K(T)=A\exp(-\frac{Ea}{RT})$$

where Ea is the activation energy, *R* is the gas constant, *A* is the pre-exponential factor, and T is the temperature. The kinetic parameters during the nickel oxidation are listed in Table 1. Solving Equation (3) to obtain the conversion of Ni to NiO with time at different temperatures is shown in Figure 3. The oxidation reaction rate in some studies [34,35] is slower than the parameters listed in Table 1. Therefore, the oxidation times given in this study are on the conservative side.

### 3.3. Thermo-Mechanical Model

The FE model imported into Abaqus contained 459,318 nodes and 2,499,970 tetrahedral meshes (Type C3D4). The convergence of the finite element simulations is directly related to the number and type of meshes. When the number of elements of the Ni-YSZ microstructure exceeds 160,000, the thermal stress values obtained from the simulations are consistent [37]. Meanwhile, the quality of the mesh (the aspect ratio, warpage, and skewness) also affects the simulation results. This FE model was mesh-verified without error meshes. The mechanical parameters of the Ni/NiO and 8YSZ used in the model are summarized in Table 2. In the model simulation, the Ni/NiO and 8YSZ within the electrodes are isotropic and homogeneous and comply with the theory of linear elasticity; the reference temperature is set at 20 °C, and the operating temperatures are 600, 700, and 800 °C. The modeling of the interaction between the Ni and 8YSZ was set up as a general contact to enhance the robustness of the complex microstructures. In addition, the stress analysis was run using the Abaqus implicit finite element solver.

The roller support boundary conditions were set on the YZ plane with X = 0, the XY plane with Z = 0, and the XZ plane with Y = 0 within the RVEs. These boundary conditions ensured that the vertical displacement of the RVEs along the surface was equal to zero.

## 4. Results and Discussion

### 4.1. Stress Distribution in Microstructures

**Figure 4:**
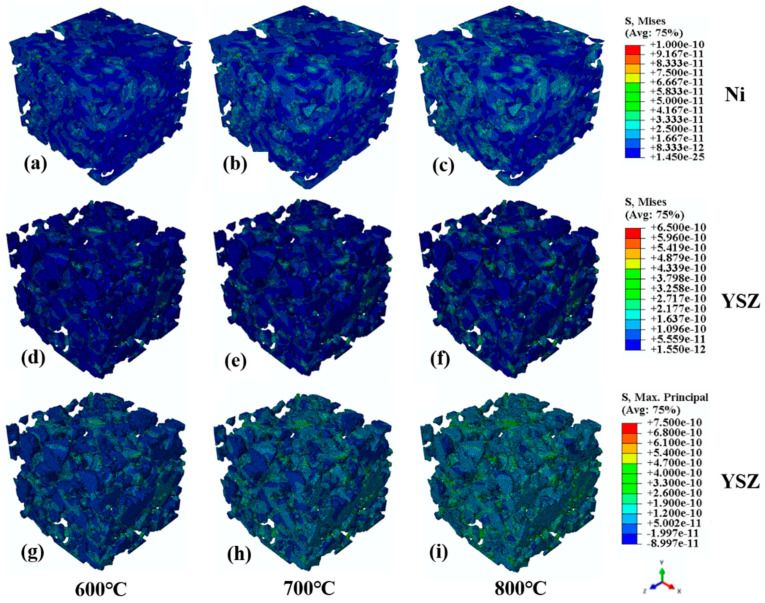
The von Mises stresses and maximum principal stresses of the reconstructed Ni-8YSZ anode microstructures. (**a**) von Mises stress of the nickel phase at 600 °C; (**b**) von Mises stress of the nickel phase at 700 °C; (**c**) von Mises stress of the nickel phase at 800 °C; (**d**) von Mises stress of the 8YSZ phase at 600 °C; (**e**) von Mises stress of the 8YSZ phase at 700 °C; (**f**) von Mises stress of the 8YSZ phase at 800 °C; (**g**) Maximum principal stress of the 8YSZ phase at 600 °C; (**h**) Maximum principal stress of the 8YSZ phase at 700 °C; (**i**) Maximum principal stress of the 8YSZ phase at 800 °C.

Figure 4 displays the von Mises stresses and maximum principal stresses of the reconstructed Ni-8YSZ anode microstructures at 600 °C, 700 °C, and 800 °C. Figure 4a–c show the von Mises stresses of the nickel phase in the anode at normal operating temperatures of 600 °C, 700 °C, and 800 °C, respectively. The unit output in Abaqus is N·nm^−2^, which is converted to MPa in the subsequent analysis. Based on the simulation results, it can be seen that the stress values of the nickel phase rise with the increase in the operating temperature, and the maximum von Mises stresses are 69.6 MPa, 79.3 MPa, and 90.9 MPa. Figure 4d–f indicate the von Mises stresses of the 8YSZ phase at the operating temperature, and it can be seen that the 8YSZ is subjected to thermal mismatch stresses of up to 430.6 MPa, 522.5 MPa, and 634.8 MPa. The relatively high stresses are distributed on the restraining surfaces and the contact surfaces with the 8YSZ. Nickel is regarded as a soft material at high-temperature conditions and its modulus of elasticity is minimal; therefore, the Ni phase suffers significantly lower stresses than the 8YSZ. Figure 4g–i demonstrate the maximum principal stresses of the 8YSZ at different operating temperatures, which reached 460.4 MPa, 566.2 MPa, and 686.9 MPa, respectively. Since 8YSZ is a typical brittle material at high temperatures [41], it is extremely sensitive to tensile stresses, and its fracture can be determined by the maximum tensile stress theory. At the thin necks, at the thin walls of the 8YSZ, and at the sharp contacts with the Ni phase, the maximum tensile stress reaches the fracture stress $\sigma_{f}$ of the material at the operating temperature. Previous studies [42,43] also revealed through experimental studies that the fracture region of the Ni-8YSZ anodes occurred on the 8YSZ skeleton during operation. In addition, when nickel is distributed on the exterior of the 8YSZ, it could potentially mitigate the influence of oxidative expansion on the 8YSZ in the opposite direction.

**Figure 5:**
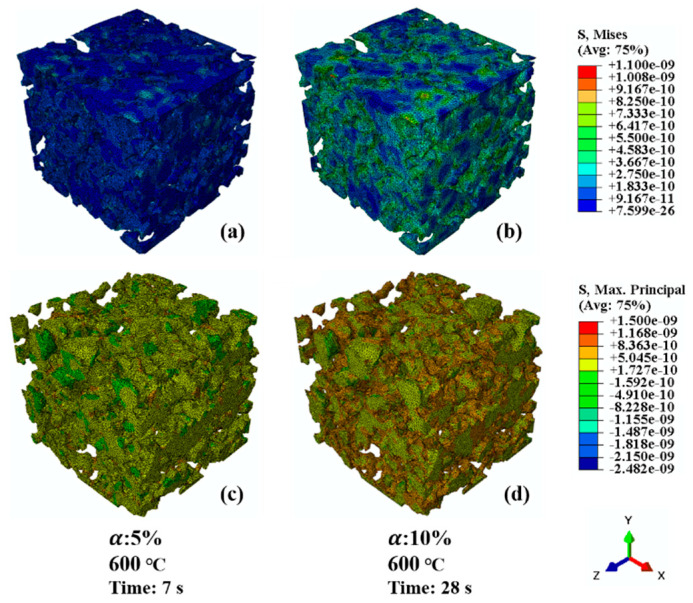
The von Mises stress distribution in the nickel phase and the maximum principal stress in the 8YSZ phase under re-oxidation. (**a**) von Mises stress for 5% oxidation of nickel at 600 °C; (**b**) von Mises stress for 10 % oxidation of nickel at 600 °C; (**c**) Maximum principal stress for 5% oxidation of nickel at 600 °C; (**d**) Maximum principal stress for 10% oxidation of nickel at 600 °C.

Figure 5 shows the stress distribution of the Ni-8YSZ anode microstructures subjected to both re-oxidation and thermal mismatch. It can be seen that, when the degree of oxidation reaches 5%, there is a fraction of the region of the nickel/nickel oxide where the von Mises stress of 438.6 MPa exceeds the yield strength of the material of 395 MPa and enters into the plastic deformation, which is probably one of the reasons for the irreversible deformation under anodic redox cycling. Since the model only considers the elastic deformation, the maximum von Mises stress reaches 900 MPa. Figure 5c,d display the stress distribution of the max first principal stress in the 8YSZ phase for 5% and 10% nickel oxidation. It is fatal for the 8YSZ phase skeleton in the microstructure once the nickel is oxidized, with most of the region under high tensile stresses, and the sharp and thin-necked portions are able to reach 0.8 to 1.5 GPa. According to the simulation results, it is feasible that part of the 8YSZ skeleton has already fractured at the early stage of oxidation, which affects the subsequent morphology evolution of the microstructure. It requires 4D in situ visualization for further confirmation. In addition, the fracture of the 8YSZ after the initial period of nickel oxidation could increase the triple-phase boundary (TPB) density, thus enhancing the electrochemical performance.

**Figure 6:**
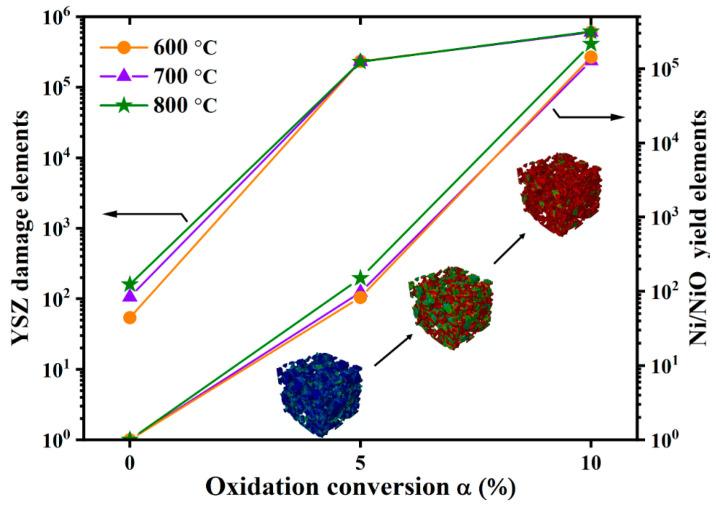
Damage and yield elements of the Ni-8YSZ microstructure under reoxidation.

Figure 6 demonstrates the yield of nickel and damage of the 8YSZ phases at different reoxidation conversions, with the nickel phase being a criterion by the theory of maximum torsional (shearing) strain energy, and the 8YSZ phase being determined to be damaged through the maximum tensile stress theory. Within the RVE, the red portions represent the areas that might be damaged and yielded. It can be noticed that the red areas increase significantly as the degree of oxidation increases and develops from the initial sharp and thin necks to the main contact surfaces. Under normal operating conditions, the Ni-8YSZ microstructure suffers thermal stress. In this case, the nickel phase was sufficiently safe, whereas 54, 106, and 160 elements in the 8YSZ phase were damaged at operating temperatures of 600, 700, and 800 °C, respectively. These elements are concentrated in the thin necks and the relatively sharp contact surfaces of the two phases. At the 5% oxidation stage, when the von Mises stress in the nickel has approached the critical criterion, the yielded elements in the nickel are 83, 97, and 150, respectively. Meanwhile, 16.17% of the elements in the 8YSZ have been damaged, which indicates that microcracks might have already occurred in portions of the skeleton. When the degree of oxidation reaches 10%, 12.06–20.37% of the elements in the nickel phase have reached the yield point. A total of 41.33% of the elements in the 8YSZ phase are damaged.

### 4.2. Model Validation

The Ni-8YSZ microstructure model is validated and compared with the thermal stress results of similar SOFCs in the cited references. The results are subject to deviations due to differences in the boundary conditions and reference temperature, but these deviations are within the acceptable limits. Therefore, the comparison of the results is accompanied by remarks on the conditions, and all of the configurations are listed in Table 3.

### 4.3. Failure Probability Analysis

Since the 8YSZ functions as the predominant support structure for the Ni-8YSZ, it is important to predict its probability of failure under reoxidizing conditions. The Weibull method of brittle material failure analysis was used to evaluate the probability of the mechanical failure of all 8YSZ elements under reoxidation conditions [23,44]. The mechanical failure probability $P_{fail}$ for each element on the 8YSZ skeleton is defined as follows:

(5) $$P_{fail}=1-\prod_{i-1}^{3}\exp(-{\int_{V}\left(\frac{\sigma_{i}}{\sigma_{w}}\right)}^{m}\frac{dV}{V_{0}})$$

where $\sigma_{w}=282$ MPa is the Weibull characteristic strength of the material under high temperature, and the constant *m* = 8 is the Weibull modulus. *V*_0_ = 0.27 mm^3^ is the 8YSZ reference volume used in the $\sigma_{w}$ measurement [45], and *V* is the volume of each element in the 8YSZ phase. $\sigma_{i}$ ($\sigma_{1}>\sigma_{2}>\sigma_{3}$) are the three principal stresses. Due to the Weibull method only considering tensile stresses, if $\sigma_{i}$ < 0 (compressive stresses), the $\sigma_{i}$ is set to zero in the calculation.

**Figure 7:**
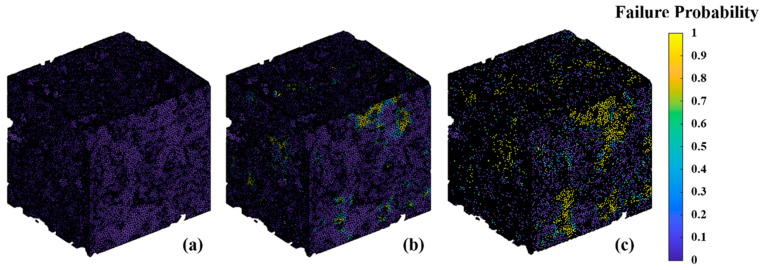
Failure probability of the 8YSZ skeleton under different reoxidation conversions. (**a**) Reoxidation conversion = 0; (**b**) Reoxidation conversion = 5%; (**c**) Reoxidation conversion = 10%.

Figure 7 demonstrates the failure probability of the 8YSZ skeleton at 600 °C with different reoxidation conversions. It can be clearly seen that, as the reoxidation conversion increases, the failure probability elevates significantly and the area of the failure region expands. As shown in Figure 7a, when the nickel phase is not oxidized at 600 °C, the failure probability of the 8YSZ skeleton can reach up to 0.47 at the thin necks and sharp corners. There are only seven elements with a failure probability higher than one-tenth, and the other elements are in a fairly safe condition. Figure 7b shows that, at a 5% reoxidation conversion, the failure probability of the 8YSZ skeleton at the necks and corners almost reaches one. At some contact surfaces between the nickel and 8YSZ, the failure probability also approaches 0.2–0.5. With the oxidation reaction time reaching 28 s and the degree of reoxidation at 10%, as it is shown in Figure 7c, the majority of the 8YSZ skeleton area is in contact with nickel, and the probability of failure is one. This indicates that less than half a minute after air leakage into the anode support layer, the area of the 8YSZ skeleton in contact with the nickel has been damaged. There are experimental results obtained that show that the fracture stress of 8YSZ at high temperatures is elevated to approximately 400 MPa, and the Weibull modulus m = 11 [41]. Therefore, the predicted failure probability of the 8YSZ under reoxidized conditions in this study is higher, which is a relatively conservative prediction. If reoxidation continues, it ultimately induces a rearrangement of the entire 8YSZ skeleton. The potential mitigation strategies include the following: oxygen detection alarms can be installed in the inlet channels of the anode, and the beginning of the reoxidation can be detected in a timely manner. Afterwards, the air valve can be quickly closed to stop the continuous leakage of oxygen, thus reducing the oxygen concentration in the inlet channel. Lastly, the operating temperature can be appropriately dropped (400–500 °C) [46], not only to ensure the material strength, but also to increase the activation energy of the reaction to reduce the rate of the reoxidation reaction. These methods maximize the integrity of the microstructure and prevent 8YSZ fractures in the microstructure.

## 5. Conclusions

In this work, a numerical finite element analysis of the reoxidation and thermal expansion of the Ni-8YSZ anode of a solid oxide fuel cell has been investigated at the microscopic level. Thermal stresses due to the thermal mismatch between the Ni and 8YSZ phases in the electrode and oxidative stresses arising from the reoxidation of nickel caused by the leakage of the sealing device or a high gas utilization efficiency mean that the anode could be suffering from the normal operation. The key results of this study are as follows: when the microstructures are subjected to thermal mismatch stresses at different operating temperatures, they operate in a relatively safe condition. Once oxidation occurs, a small portion of the nickel/nickel oxide yields, and the tensile stresses on the 8YSZ crack at the thin neck/sharp area when the reoxidized degree is at 5%. When the oxidation extent reaches 10%, there is a high probability of mechanical failure of the 8YSZ at the major contact surfaces, resulting in damage to the electrode. The effect of oxidative expansion on the 8YSZ could be mitigated by appropriately increasing the porosity and by distributing the nickel towards the outside of the 8YSZ during the manufacturing process. This research could provide insights into the optimized design of SOFC electrode microstructures for antioxidants, which contributes to the enhancement of the mechanical stability and operating life of the SOFC under long-term operation.

## Figures and Tables

**Table 1:** Kinetic parameters of nickel oxidation at different temperatures [36].

	600 °C	700 °C	800 °C
K(T) (m·s^−1^)	9.14 × 10^−5^	2.25 × 10^−4^	5.00 × 10^−4^
Ea (kJ·mol^−1^)	65.0	70.6	75.4

**Table 2:** Mechanical parameters of the anode components [38,39,40].

	E (GPa)			Possion’s Ratio ν	TEC α (10^−6^·K^−1^)	Yield/Fracture Stress (MPa)		
8YSZ	600 °C	700 °C	800 °C	0.313	10.5	600 °C	700 °C	800 °C
	126.9	132.2	141.7			395.3	412.4	433.2
Ni	5.9			0.310	13.5	–		
NiO	600 °C	700 °C	800°C	0.330	11.8	395		
	104.9	107.4	110					

**Table 3:** Comparison of the simulation results for the Ni-8YSZ against the literature data.

	This Study	Celik et al. [37]	Clague et al. [22]
Boundary condition	Roller support at three planes	–	Point constraint and roller support of one cutting surface
Reference temperature	Room temperature	Room temperature	Room temperature
Size (µm)	6.40 × 6.40 × 6.40	10 × 10 × 10	6.68 × 5.04 × 1.50
Mesh quantity	2,499,970	300,000	285,000
Stress (MPa)	69.6–90.9	40	≥59

## Data Availability

The data that support the findings of this study are available from the corresponding author upon reasonable request.
